# French Commission of Lunacy

**Published:** 1844-11-02

**Authors:** 


### FRENCH COMMISSION OF LUNACY.

In France, when a person has been arrested for any crime, of which it is supposed he is not morally guilty, in consequence of insanity, the point is determined by a commission of medical men, and not by a court of law. The process, in essence, is as follows. The physician of the prison, together with the individuals composing the commission, who are conversant with all the phases of insanity, give the closest possible attention to all the circumstances of the case, and carefully analyze the criminal's deportment, general character, habits, propensities, education, lineage, &c., together with the object to be gained or feeling to be gratified in the perpetration of the offence of which he stands charged. When they are perfectly satisfied in regard to all those points, a report is made to the proper tribunal; and if the commissioners decide that the prisoner is insane, he is placed in a lunatic institution, and no further cognizance is taken of the matter by government. On the other hand, should the report show that the offender was sane at the period when the act for which he is incarcerated was committed, then he abides all the pains and penalties of a violated law.

After the practical advantages of this system have been thoroughly proved by the test of nearly twenty years in France, it recommends itself to the wise and humane consideration of legislators in the United States. We ought to profit by the experience of all civilized countries, and whatever promises to promote human happiness, even if it contemplates the physical condition of the most wretched class of criminals, should engage the profound consideration of men in all conditions of society.—*Boston Med. & Sur. Journal.*

